# SARS-CoV-2 Infection Dysregulates Cilia and Basal Cell Homeostasis in the Respiratory Epithelium of Hamsters

**DOI:** 10.3390/ijms23095124

**Published:** 2022-05-04

**Authors:** Tom Schreiner, Lisa Allnoch, Georg Beythien, Katarzyna Marek, Kathrin Becker, Dirk Schaudien, Stephanie Stanelle-Bertram, Berfin Schaumburg, Nancy Mounogou Kouassi, Sebastian Beck, Martin Zickler, Gülsah Gabriel, Wolfgang Baumgärtner, Federico Armando, Malgorzata Ciurkiewicz

**Affiliations:** 1 Department of Pathology, University of Veterinary Medicine Hanover Foundation, 30559 Hanover, Germany; tom.schreiner@tiho-hannover.de (T.S.); lisa.allnoch@tiho-hannover.de (L.A.); georg.beythien@tiho-hannover.de (G.B.); katarzyna.marek@tiho-hannover.de (K.M.); kathrin.a.becker@basf.com (K.B.); federico.armando@tiho-hannover.de (F.A.); malgorzata.ciurkiewicz@tiho-hannover.de (M.C.); 2 Center for Systems Neuroscience (ZSN), University of Veterinary Medicine Hanover Foundation, 30559 Hanover, Germany; 3 Fraunhofer Institute for Toxicology and Experimental Medicine, 30625 Hanover, Germany; dirk.schaudien@item.fraunhofer.de; 4 Department for Viral Zoonoses-One Health, Leibniz Institute for Experimental Virology (HPI), 20251 Hamburg, Germany; stephanie.stanelle-bertram@leibniz-hpi.de (S.S.-B.); berfin.schaumburg@gmail.com (B.S.); nancy.mounogou@leibniz-hpi.de (N.M.K.); sebastian.beck@leibniz-hpi.de (S.B.); martin.zickler@leibniz-hpi.de (M.Z.); guelsah.gabriel@leibniz-hpi.de (G.G.)

**Keywords:** SARS-CoV-2, COVID-19, trachea, golden Syrian hamster, respiratory epithelium, cilia, histology, immunohistochemistry, scanning electron microscopy, transmission electron microscopy

## Abstract

Similar to many other respiratory viruses, SARS-CoV-2 targets the ciliated cells of the respiratory epithelium and compromises mucociliary clearance, thereby facilitating spread to the lungs and paving the way for secondary infections. A detailed understanding of mechanism involved in ciliary loss and subsequent regeneration is crucial to assess the possible long-term consequences of COVID-19. The aim of this study was to characterize the sequence of histological and ultrastructural changes observed in the ciliated epithelium during and after SARS-CoV-2 infection in the golden Syrian hamster model. We show that acute infection induces a severe, transient loss of cilia, which is, at least in part, caused by cilia internalization. Internalized cilia colocalize with membrane invaginations, facilitating virus entry into the cell. Infection also results in a progressive decline in cells expressing the regulator of ciliogenesis FOXJ1, which persists beyond virus clearance and the termination of inflammatory changes. Ciliary loss triggers the mobilization of p73^+^ and CK14^+^ basal cells, which ceases after regeneration of the cilia. Although ciliation is restored after two weeks despite the lack of FOXJ1, an increased frequency of cilia with ultrastructural alterations indicative of secondary ciliary dyskinesia is observed. In summary, the work provides new insights into SARS-CoV-2 pathogenesis and expands our understanding of virally induced damage to defense mechanisms in the conducting airways.

## 1. Introduction

A novel pandemic betacoronavirus, severe acute respiratory syndrome coronavirus 2 (SARS-CoV-2), responsible for the coronavirus disease 2019 (COVID-19), is responsible for over 500 million infections and over 6.2 million fatal outcomes to this day (April 2022) [1]. SARS-CoV-2 replication is generally confined to the upper and lower respiratory tract, and the epithelial cells covering the conducting airways represent the main initial target of the virus [2,3,4]. The respiratory epithelium is mainly composed of ciliated cells and nonciliated secretory goblet cells [5,6]. These cells work in synergy to provide the main innate defense mechanism of the airways, mucociliary clearance [5,6,7,8]. This depends on two essential entities: (i) mucus production by secretory cells for the entrapment of inhaled particles and (ii) continuous evacuation of mucus and particles from the airways by synchronized movement of respiratory cilia [4]. Damage to the respiratory epithelium results in inefficient mucociliary clearance, which is commonly observed in several respiratory viral, bacterial, and fungal infections as well as in noninfectious respiratory diseases [4,9,10,11,12,13]. The importance of a functioning mucociliary apparatus is underlined by observations from genetic respiratory disorders like Cystic Fibrosis (CF) and Primary Ciliary Dyskinesia (PCD), diseases that are characterized by the malfunction of mucus production and cilia function, respectively. Affected patients are predisposed to chronic respiratory infections and frequently develop irreversible sequelae like bronchiectasis [6,14,15,16].

Several respiratory viruses, including common cold coronaviruses (e.g., HCoV-E229, HCoV-OC43), as well as rhinovirus and influenza have a tropism for ciliated cells and compromise mucociliary clearance [13,17]. More recently emerged viruses, e.g., SARS-CoV-1 and Middle East Respiratory syndrome-coronavirus (MERS-CoV), are also known to cause ciliary loss [18,19]. The pathomechanisms of virus-induced ciliary function impairment are highly virus-specific, and many viruses have developed ingenious methods to harm cilia. For instance, SARS-CoV-1, MERS-CoV, as well as the Influenza virus and respiratory enterovirus damage the local defenses through an aggressive approach, i.e., by inducing cell death of the respiratory epithelium. Other respiratory viruses, like HCoV-OC43 and HCoV-229E, use more subtle methods like the inhibition of pathways critical for ciliogenesis and cilia maintenance, which results in ciliary loss but otherwise mild damage to the epithelium [4,13,18,20,21]. Because of their detrimental effects on ciliated cells, respiratory viruses frequently facilitate bacterial colonization of the deeper airways and translocation through the epithelial barrier, which increases the incidence and severity of secondary infections [14]. In addition to causing a loss of cilia, respiratory viral and bacterial infections have been reported to induce ultrastructural abnormalities in motile cilia, resulting in uncoordinated ciliary movement, also known as acquired or secondary ciliary dyskinesia (SCD) [22,23,24,25]. Importantly, SCD can potentially contribute to impaired lung defenses and induce a long-lasting susceptibility to respiratory infections [25,26,27]. For instance, reduced ciliation and morphologic indicators of SCD along with a reduced ciliary beat frequency have been detected for up to 17 weeks following acute bronchiolitis in infants [28].

It is known that SARS-CoV-2 causes ciliary loss in humans and susceptible animal species, but the exact pathomechanism responsible for this is not completely understood [29,30,31,32,33]. In humans, it has been demonstrated in vitro and in vivo that infection is associated with decreased expression of the transcription factor Forkhead box protein J1 (FOXJ1) in the respiratory epithelia, which is required for the correct docking of basal bodies as well as cilia motility and length [31,34,35,36,37]. However, the exact chain of events and mode of interaction between SARS-CoV-2 and FOXJ1 remains unclear. Moreover, little is known about the regeneration of the cilia after virus clearance and the possible long-term consequences of COVID-19 in terms of mucociliary clearance. Another open question regarding the interaction of SARS-CoV-2 and the respiratory epithelium is the exact mode by which the virions enter ciliated cells. The main entry factors for SARS-CoV-2, Angiotensin-converting enzyme 2 receptor (ACE2), and Transmembrane Protease Serine 2 (TMPRSS2) are expressed in ciliated cells of the human respiratory epithelium and predominantly localize to the cilia themselves [32,38]. However, the virus has not been shown to enter the cytoplasm via the ciliary axoneme, and thus, it remains unclear how SARS-CoV-2 uses the interaction with ACE2 on the cilia to progress into the cell [39].

Some of these open questions can only be approached by using appropriate animal models of COVID-19. The golden Syrian hamster is an appropriate and well-established model for investigating many aspects of SARS-CoV-2 infection [40,41,42,43,44,45]. Importantly, ciliary loss, along with misshapen and shortened cilia after SARS-CoV-2 infection, has been described in golden Syrian hamsters at 4 days post infection [31]. Thus, the species is suitable for investigating the mechanisms and long-term consequences of SARS-CoV-2-induced ciliary loss.

The aim of the following study was to characterize the changes observed in the ciliated respiratory epithelium during a SARS-CoV-2 infection in detail. The tracheal epithelium of SARS-CoV-2-infected golden Syrian hamsters was investigated by light and transmission and scanning electron microscopy. Moreover, immunohistochemical expression of activated caspase-3, FOXJ1, p73, and CK14 within the tracheal respiratory epithelium was investigated to provide new insights into the dysregulation of the cellular homeostasis of the respiratory epithelium after SARS-CoV-2 infection.

## 2. Results

### 2.1. SARS-CoV-2 Transiently Infects the Tracheal Epithelium of Golden Syrian Hamsters

**Figure 1:**
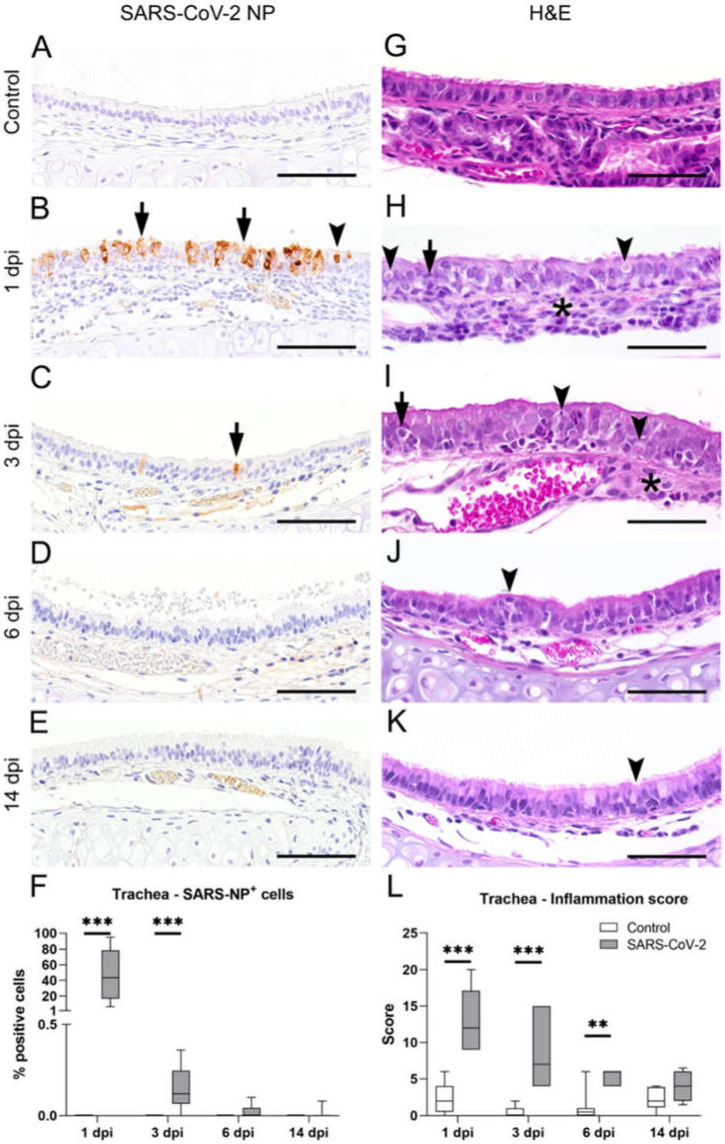
Characterization of inflammatory alterations and virus load in the trachea of severe acute respiratory syndrome coronavirus 2 (SARS-CoV-2)-infected hamsters. Representative images of tracheal sections from SARS-CoV-2-infected and control animals, immunolabeled with a SARS-CoV-2 nucleoprotein (NP) antibody (**A**–**E**) or stained with hematoxylin and eosin (H&E) (**G**–**K**) at 1, 3, 6, and 14 days post infection (dpi), respectively. (**A**) Control animals showed no positive staining for SARS-CoV-2 NP. (**B**) A high amount of the NP^+^ signal was detected at 1 dpi in the cytoplasm and nucleus of ciliated (arrowhead in (**B**)) and nonciliated cells (arrows in (**B**)). (**C**) Only single cells showed the NP^+^ signal (arrow in (**C**)) in SARS-CoV-2-infected animals at 3 dpi. (**D**,**E**) A positive signal was only rudimentarily observed in SARS-CoV-2-infected animals at 6 and 14 dpi. (**F**) Quantification of SARS-CoV-2 NP^+^ cells showed the highest amount of NP^+^ cells at 1 dpi and a significantly higher amount of NP^+^ cells at 3 dpi compared with control animals. (**G**) H&E stained tracheal sections of control animals showed an intact, columnar, pseudostratified, ciliated epithelium. (**H**) At 1 dpi, the tracheal epithelium of SARS-CoV-2-infected animals was composed of moderate to severe, subepithelial, heterophilic, and lymphohistiocytic inflammation (asterisk in (**H**)) with rare apoptotic cells (arrowheads in (**H**)) and mild to moderate exocytosis of heterophils (arrow in (**H**)). (**I**) At 3 dpi, SARS-CoV-2-infected animals showed moderate, subepithelial, heterophilic, and lymphohistiocytic inflammation (asterisk in (**I**)) with low numbers of apoptotic figures (arrowheads in (**I**)) and mild to moderate exocytosis of heterophils (arrow in (**I**)). (**J**,**K**) Epithelial hyperplasia could be observed at 6 and 14 dpi (arrowhead in (**J**,**K**)) in the trachea of SARS-CoV-2-infected animals. (**L**) Inflammation in the trachea was assessed using a semiquantitative scoring system, including the extent and severity of inflammation (for details, see material and methods: *4.2 Histology*). The semiquantitative evaluation revealed significantly higher scores in SARS-CoV-2-infected animals at 1, 3, and 6 dpi. (**F**,**L**): Box and whisker plots show the medians, quartiles and ranges. Asterisks indicate significant differences * *p* < 0.05, ** *p* < 0.01, *** *p* < 0.001, Mann–Whitney-U test. (**A**–**E**,**G**–**K**): Bars: 50 µm.

Golden Syrian hamsters were intranasally inoculated with a suspension with 10^5^ PFU SARS-CoV-2 or phosphate-buffered saline (PBS, control) and euthanized at 1, 3, 6, or 14 dpi. At first, formalin-fixed and paraffin embedded (FFPE) tracheal tissue sections were examined for intraepithelial SARS-CoV-2 nucleoprotein (NP) immunolabeling to confirm infection and to determine the cellular distribution of the virus in SARS-CoV-2-infected animals. Control animals did not show any SARS-CoV-2 NP immunolabeled cells at any time point (Figure 1A). In SARS-CoV-2-infected hamsters, immunolabeling was restricted to the epithelium and included ciliated as well as nonciliated cells (Figure 1B–E). Infection was widespread at 1 dpi in most animals, with a group median of 40% infected cells (Figure 1F). At 3 dpi, only occasional immunolabeled cells, encompassing less than 1% of the total epithelium, were observed. Virus clearance was completed in most hamsters at 6 and 14 dpi. After confirming the successful infection and subsequent virus elimination, we analyzed the SARS-CoV-2-induced histopathological changes.

On H&E stained sections from control animals, the epithelium was largely intact and showed dense ciliation (Figure 1G). SARS-CoV-2-infected hamsters showed moderate to severe, multifocal to coalescing, subepithelial infiltration with heterophils, macrophages, and lymphocytes and mostly heterophilic exocytosis. Qualitative histopathological analysis of the samples revealed that SARS-CoV-2 infection resulted only in occasional cell death, but there was widespread loss of cilia, which was most prominent at 3 dpi. At 14 dpi, it appeared that normal ciliation was restored when compared with the control animals. In addition, starting from 3 dpi, the epithelium showed a hyperplastic appearance, characterized by the piling up of cells and occasional mitotic figures (Figure 1H–K). Inflammation was assessed with a semiquantitative score that revealed significantly increased inflammation in SARS-CoV-2-infected animals compared with those in the control group at 1 dpi (median score control: 2; median score SARS-CoV-2: 12; *p* < 0.001), 3 dpi (median score control: 0; median score SARS-CoV-2: 7; *p* < 0.001), and 6 dpi (median score control: 0.5; median score SARS-CoV-2: 6; *p* = 0.003), as shown in Figure 1L.

Taken together, histopathology and immunolabeling revealed that SARS-CoV-2 infection causes transient tracheitis with virus clearance within one week and the termination of inflammation within two weeks.

### 2.2. Trachea of SARS-CoV-2-Infected Hamsters Shows Transient Ciliary Loss Peaking at Day 3 Post-Infection, Followed by Apparent Cilia Regeneration within Two Weeks

**Figure 2:**
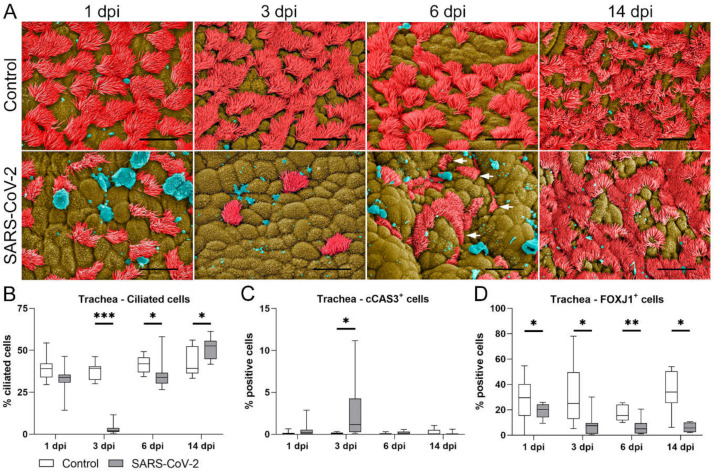
SARS-CoV-2 causes transient ciliary loss followed by cilia regeneration within two weeks, and this is accompanied by the prolonged reduction of FOXJ1^+^ cells. (**A**) Scanning electron microscopic (SEM) findings in the tracheal luminal epithelium. Representative SEM images of the tracheal epithelium of control and SARS-CoV-2-infected animals euthanized at 1, 3, 6, and 14 days post-infection (dpi). Cilia are colored in red, nonciliated areas are in brown, and cellular debris, mucus and unidentifiable material are in blue. Bars: 10 µm. In control animals, the epithelial surface showed ciliated cells with long cilia covering approximately 50% of the surface. In SARS-CoV-2-infected animals, there was mild reduction of ciliated cells at 1 dpi and a marked reduction at 3 dpi. At 6 dpi, ciliated cells were partly restored, but cilia appeared shortened (arrows). At 14, no differences were observed between control and SARS-CoV-2-infected animals. (**B**) Quantification of ciliated cells. At 3 and 6 dpi, SARS-CoV-2-infected animals showed significantly fewer ciliated cells compared with controls. (**C**) Quantification of cCAS3^+^ cells showed a mildly elevated percentage of apoptotic cells at 3 dpi. (**D**) Quantification of FOXJ1 showed a significant decrease in the percentage of FOXJ1^+^ cells in SARS-CoV-2-infected animals compared with controls at 1, 3, 6, and 14 dpi. B, C, D: Box and whisker plots with medians, quartiles, and ranges. Asterisks indicate significant differences: * *p* < 0.05, ** *p* < 0.01, *** *p* < 0.001, Mann–Whitney-U-test.

Subsequently, we performed scanning electron microscopy (SEM) on trachea samples in order to verify and quantify the ciliary loss detected by light microscopy (Figure 2A,B). At all investigated timepoints, the tracheal surface of control hamsters showed a random and patchy distribution of ciliated cells, as previously described for this species [46]. In SARS-CoV-2-infected animals, the overall number of ciliated cells was reduced. (Figure 2A). At 1 dpi, SARS-CoV-2-infected animals showed mild reduction of ciliated cells compared with the control animals (median control: 39.02%; median SARS-CoV-2: 33.80%), while an almost complete loss of cilia was observed at 3 dpi (median control: 39.29%; median SARS-CoV-2: 1.85%, *p* < 0.001). Interestingly, ciliation was partly restored in SARS-CoV-2-infected hamsters at 6 dpi, although cilia were still reduced in number or appeared shortened when compared with the long cilia covering the epithelial surface of the control animals (see arrows in Figure 2A,B, median control: 41.99%; median SARS-CoV-2: 33.77%; *p* = 0.006). At 14 dpi, ciliation was apparently restored, and SARS-CoV-2-infected animals even showed a significantly higher number of ciliated cells compared with the control animals (median control: 39.15%; median SARS-CoV-2: 52.53%; *p* = 0.018). It is important to note that the vast majority of partly or completely deciliated cells, observed at 1 and 3 dpi, still appeared intact, and that SEM analysis showed no evidence of significant cytolysis or exfoliation of the epithelium. In order to confirm this observation, we investigated the cell death rate using cleaved caspase-3 (cCAS-3) immunolabeling. The quantification of positive cells revealed a mild but significant increase in the number of apoptotic cells at 3 dpi in the SARS-CoV-2-infected group when compared with the control animals (Figure 2C, median control: 0.09%; median SARS-CoV-2: 1.15%; *p* = 0.001, see also Supplementary Figure S1A). However, the number of immunolabeled cells was below 5% in most samples, and therefore, the rate of apoptosis could not entirely account for the extent of ciliary loss.

In summary, SARS-CoV-2 infection is followed by a severe loss of cilia, peaking at 3 dpi, which seems not to be mainly caused by death or the sloughing of ciliated cells. Starting from 6 dpi, cilia regenerate, and baseline levels are apparently restored after two weeks. However, the regrown cilia partly show morphologic alterations. In the next step, mechanisms of ciliary loss were investigated.

### 2.3. SARS-CoV-2-Induced Ciliary Loss Is Accompanied by a Decreased Number of FOXJ1^+^ Cells and Cilia Internalization Colocalizing with Virus Entry

It was previously shown that ciliary loss is associated with the loss of FOXJ1 mRNA and protein expression in human bronchial cell cultures infected with SARS-CoV-2 [31]. FOXJ1 expression has not been investigated in the hamster model yet. Therefore, we performed an immunohistochemical analysis to detect and quantify the number of FOXJ1-positive epithelial cells (Figure 2D, Supplementary Figure S1B) in hamster trachea. Starting from 1 dpi, SARS-CoV-2-infected hamsters displayed a significantly lower number of FOXJ1^+^ cells compared with members of the control group (median control: 30.24%; median SARS-CoV-2: 20.13%; *p* = 0.047). Interestingly, SARS-CoV-2-infected animals were shown to have an ongoing loss of FOXJ1^+^ cells with significantly lower percentages of these cells compared with the control group at 3 dpi (median control: 24.97%; median SARS-CoV-2: 7.42%; *p* = 0.006), 6 dpi (median control: 15.54%; median SARS-CoV-2:4.98%; *p* = 0.004), and 14 dpi (median control: 31.60%; median SARS-CoV-2: 4.44% *p* = 0.020) as shown in Figure 2D.

**Figure 3:**
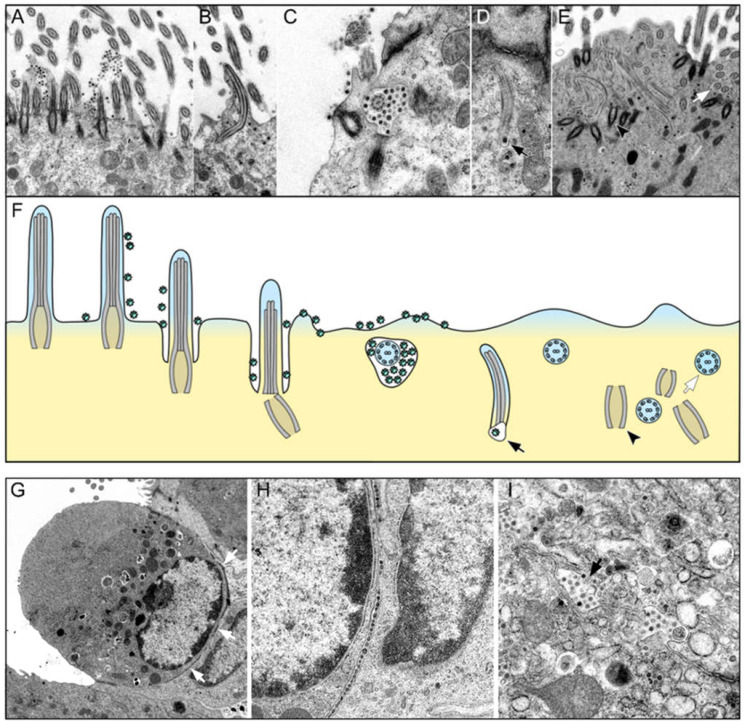
Cilia retraction and virus entry and spread in the tracheal epithelium of SARS-CoV-infected hamsters at 1 day post infection (dpi). (**A**–**F**) Representative transmission electron micrographs showing different stages of ciliary retraction and a schematic illustration of the proposed sequence of cilia internalization and SARS-CoV-2 entry into the cell. (**A**) Numerous virions are attached to the cilia and the microvilli surface. (**B**) Retraction of an entire cilium into the cytoplasm. Virus particles are in close contact with the retracted axoneme. (**C**) Cross-section of a completely internalized ciliary axoneme surrounded by virus particles and an infolding of the plasma membrane. (**D**) Internalized cilium with an associated virus particle located in a vacuole (arrow in (**D**,**F**)). (**E**) Numerous separated axonemes (white arrows in (**E**,**F**)) and basal bodies (black arrowheads in (**E**,**F**)) within the cytoplasm. Magnifications are 12,500× in (**A**,**B**), 31,500× in (**C**,**D**), and 10,000× in (**E**). (**F**) Schematic illustration of proposed sequence of cilia internalization and SARS-CoV-2 entry into the cell. (**G**–**I**) Virus spread between adjacent cells in the trachea of a SARS-CoV-2-infected hamster at 1 dpi. (**G**) SARS-CoV-2-infected epithelial cell with virus particles aligned along the basal cell pole (white arrows). (**H**) Higher magnification of G showing the basal cell pole. Virus particles lined up at the intercellular space and in the cytoplasm of the underlying cell. (**I**) Virus spreading from the intercellular space into a neighboring cell (arrow). Magnifications: 6300× in (**G**), 16,000× in (**H**), and 12,500× in (**I**).

In the next step, we hypothesized that the loss of FOXJ1 could lead to the retraction of cilia, and this could be a viral mechanism to allow particles to gain entry into the cells. Therefore, we performed TEM and analyzed the morphological features of cilia loss and the distribution of viral particles during acute infection (Figure 3A–F).

The qualitative TEM analysis of samples from SARS-CoV-2 infected hamsters euthanized at 1 dpi revealed numerous virions attached to the surface of the cilia and microvilli of the tracheal epithelium (Figure 3A). Importantly, the affected ciliary axonemes appeared structurally intact, and no fusion of virions with the axoneme membrane was observed. Thus, virions did not appear to enter the cell via this structure. However, a retraction of the entire cilium into the cytoplasm of infected cells was observed at the same time-point (Figure 3B), which co-occurred with the entry of virions adhering to the ciliary surface into the cytoplasm, as shown in Figure 3C,D. On the cross-sections, we observed internalized axonemes located in the apical cytoplasm surrounded by a vacuole, which most likely represented an infolding of the plasma membrane. Virions were frequently observed between this vesicle membrane and the axoneme. Deeper within the cell, axonemes separated from basal bodies or were found freely and without a surrounding membrane within the cytoplasm (Figure 3E). These were also often accompanied by adjacent viral particles. Disordered and internalized basal bodies were also frequently observed within the cytoplasm (Figure 3E). Interestingly, TEM investigations during the acute phase of SARS-CoV-2 infection in hamster tracheas also revealed linear arrays of virus particles within the basolateral intercellular space (Figure 3G–I). In these regions, focal discontinuities in the cell membrane and the entry of virus particles into neighboring cells were observed. These findings suggest that SARS-CoV-2 can also spread from cell to cell on the basolateral side. Of note, the majority of infected cells did not display ultrastructural features of degeneration, necrosis, or apoptosis.

In summary, immunohistochemistry revealed a significantly lower number of FOXJ1^+^ cells in SARS-CoV-2-infected hamsters. TEM analysis revealed the internalization of cilia in viable cells, which colocalized with an internalization and cell entry of virions docked at the axoneme membrane. The TEM analysis additionally revealed viral spread through the basolateral intercellular space.

### 2.4. Regenerated Cilia in the Epithelium with a Decreased Number of FOXJ1^+^ Cells Show Ultrastructural Features Indicative of Secondary Ciliary Dyskinesia

SEM investigations revealed cilia regeneration starting at 6 dpi and being completed at 14 dpi in SARS-CoV-2-infected hamsters. However, the number of FOXJ1^+^ cells did not return to baseline levels by the end of the study. Considering the importance of FOXJ1 for correct basal body docking and subsequent correct cilia formation [37,47,48], we assumed that the regrown cilia would have an abnormal ultrastructure. To confirm this assumption, we first analyzed the process of cilia restoration on the SARS-CoV-2-infected hamster tracheas by TEM investigations of hamsters euthanized at late time points.

**Figure 4:**
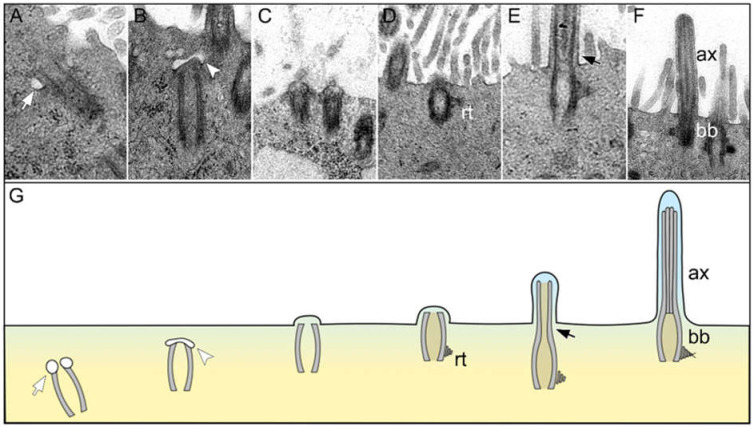
Ciliary regeneration in the trachea of a hamster 6 days after SARS-CoV-2 infection. (**A**–**G**) Representative transmission electron micrographs showing different stages of ciliary regeneration and a schematic illustration of the sequence of ciliary regeneration. (**A**) The centriole starts to produce a primary cilium by forming vesicles at the distal end (white arrows in (**A**,**G**)). (**B**) A ciliary bud develops at the distal end of the centriole invaginating the ciliary vesicle (white arrowheads in (**B**,**G**)). (**C**) The ciliary bud migrates to the apical pole of the cell and bulges the cell membrane. (**D**) The basal body develops a lateral rootlet (rt in (**D**,**G**)). (**E**) The organelle elongates apically and forms the axoneme (black arrows in (**E**,**G**)). (**F**) Mature cilia with regularly formed axoneme (ax in (**F**,**G**)) and basal body (bb in (**F**,**G**)). Magnifications are 31,500× in (**A**), 40,000× in (**B**), 10,000× in (**C**,**D**) and 12,500× in (**E**,**F**).

The first indications of ciliary regeneration were observed at 6 dpi (Figure 4A–G). Ciliogenesis began with the intracytoplasmic formation of a primary cilium showing vesicles at the distal ends of the centriole (Figure 4A). Thereafter, vesicles were invaginated by a ciliary bud (Figure 4B). Subsequently, migration and elongation of the ciliary bud and bulging onto the cell membrane were observed (Figure 4C). During the process of maturation and elongation, the ciliary bud formed a lateral rootlet (Figure 4D) and the axoneme (Figure 4E). Finally, mature cilia composed of a regularly formed axoneme and a basal body were observed (Figure 4F). However, in agreement with our SEM findings, the new cilia appeared to be shortened and blunt on longitudinal sections.

**Figure 5:**
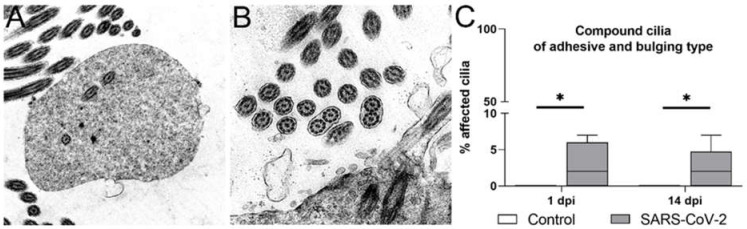
Ultrastructural abnormalities of cilia from SARS-CoV-2-infected hamsters in the tracheal epithelium before and after ciliary regeneration. (**A**,**B**) Examples of ciliary alterations observed by transmission electron microscopy of tracheal samples. The most common ultrastructural changes included compound cilia of the bulging type (**A**) and adhesive type (**B**). Magnifications are 16,000× in (**A**), 20,000× in (**B**). (**C**) The quantification of compound cilia of both types showed a significant increase in SARS-CoV-2-infected animals at 1 and 14 days post-infection (dpi). Box and whisker plots show medians, quartiles, and ranges. Asterisks indicate significant differences: * *p* < 0.05; Mann–Whitney-U-test.

Accordingly, ultrastructural alterations of axonemes were also observed in cross-sections. Observed ciliary alterations included compound cilia, ciliary blebs, as well as the disorganization of peripheral microtubules, all features indicative of secondary ciliary dyskinesia [10,13,49,50]. Compound cilia contain multiple microtubule complexes within the axoneme and can be divided into two distinct types. The bulging type is characterized by a balloon-like form with loosely and randomly distributed microtubule complexes (Figure 5A), whereas the adhesive type has a columnar shape with closely packed and parallel-oriented microtubules (Figure 5B). In compound cilia, the number of axonemes varies, but the typical 9 × 2 + 2 pattern is maintained. In contrast, a disorganized cilium is characterized by disorientation of the 9 × 2 + 2 pattern with partly peripherally arranged microtubules. A ciliary bleb describes a homogenous and unilateral bubble-like protrusion of the ciliary membrane [51,52]. Since ciliary blebs and microtubule disorganization were occasionally only observed in individual animals, we focused our analysis on compound cilia and quantified this abnormality in samples from animals euthanized during acute virial infection and at the time point of regeneration (Figure 5C). Quantification revealed significantly increased numbers of compound cilia in SARS-CoV-2-infected animals at 1 dpi (median control: 0%; median SARS-CoV-2: 2%; *p* = 0.033). Interestingly, the numbers were also significantly higher after virus clearance at 14 dpi (median control: 0%; median SARS-CoV-2: 2%; *p* = 0.041), which is in line with the decreased expression of FOXJ1.

Taken together, the ultrastructural examination of tracheal ciliated cells of SARS-CoV-2-infected hamsters confirmed the restoration of the total number of ciliated cells starting from 6 and being completed at 14 dpi, despite a lower number of FOXJ1^+^ cells. However, the higher percentage of axonemes with ultrastructural alterations in SARS-CoV-2-infected hamsters suggests that the SARS-CoV-2-induced decrease in the number of FOXJ1^+^ cells might result in secondary ciliary dyskinesia. Importantly, these alterations were also shown to be present at the time of virus elimination, indicative of a prolonged detrimental effect of SARS-CoV-2 on correct cilia formation.

### 2.5. Early Phase of SARS-CoV-2 Infection Triggers a Proliferative Response in p73^+^ and CK14^+^ Basal Cells in Hamster Trachea

Restoration of the intact ciliated respiratory epithelium after virus clearance can be achieved in two ways, either by the regeneration of sublethally injured cells with reactivation of the ciliogenesis machinery or by replacement from the basal cell pool. It was previously shown that ciliary loss in SARS-CoV-2-infected hamsters is accompanied by basal cell mobilization at 4 dpi [31]. Therefore, we subsequently examined the dynamics of basal cell populations in infected hamsters over time.

**Figure 6:**
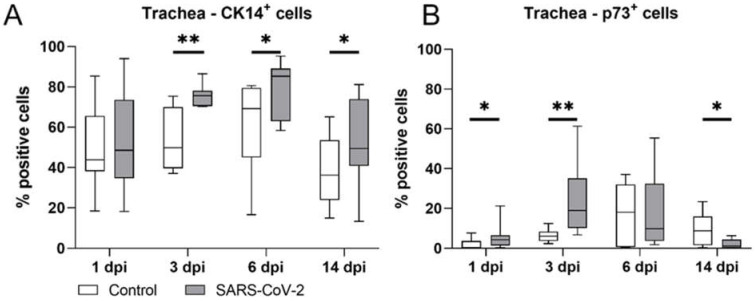
Histologic quantification of CK^+^ and p73^+^ immunolabelled cells in tracheal cross-sections. (**A**) Immunolabeling of CK14^+^ basal cells in SARS-CoV-2-infected animals revealed significant increases at 3, 6, and 14 days post-infection (dpi) in positive cells compared with controls. (**B**) Immunolabeling of p73^+^ basal cells in SARS-CoV-2-infected animals revealed significant increases in positive cells at 1 and 3 dpi and a significant decrease in positive cells at 14 dpi compared with controls. Box and whisker plots show medians, quartiles, and ranges. Asterisks indicate significant differences: * *p* < 0.05, ** *p* < 0.01, Mann–Whitney-U-test.

For this, we quantified the expression of the basal cell markers CK14 and p73 by immunohistochemistry (Figure 6, Supplementary Figure S1C,D). SARS-CoV-2-infected hamsters showed a significantly increased number of CK14^+^ cells compared with animals in the control group at 3 dpi (median control: 49.80%; median SARS-CoV-2: 75.61%; *p* = 0.001), 6 dpi (median control: 69.22%; median SARS-CoV-2: 85.27%; *p* = 0.020), and 14 dpi (median control: 36.24%; median SARS-CoV-2: 49.37%; *p* = 0.042), indicative of basal cell mobilization starting at the time of maximal ciliary loss (Figure 6A). In addition, SARS-CoV-2-infected animals showed significantly higher numbers of basal p73^+^ cells at 1 dpi (median control: 0.00%; median SARS-CoV-2: 0.04%; *p* = 0.022) and 3 dpi (median control: 0.06%; median SARS-CoV-2: 0.19%; *p* = 0.002), compared with the control animals. Subsequently, SARS-CoV-2-infected animals were shown to have a decreased number of basal p73 immunolabeled cells at 6 dpi (median control: 0.18%; median SARS-CoV-2: 0.10%), significantly lower than that found for the control group at 14 dpi (median control: 0.09%VS median SARS-CoV-2: 0.01%; *p* = 0.025) (Figure 6B).

Taken together, these findings suggest that the CK14 and p73 basal cell populations in the hamster trachea proliferate to compensate for a SARS-CoV-2-induced loss of cilia, which ceases after the restoration of ciliation at 14 dpi. However, the terminal differentiation of ciliated cells appears to be incomplete, since baseline FOXJ1 levels were not reinstated at 14 dpi.

## 3. Discussion

The present study used the golden Syrian hamster model to study tracheal epithelial changes following intranasal infection with 10^5^ plaque-forming units of SARS-CoV-2 over time. Quantification of the SARS-CoV-2 NP antigen within the epithelial cells confirmed that viral antigen clearance in the trachea was completed at 6 dpi. SARS-CoV-2 infection was shown to provoke transient tracheitis from 1 to 6 dpi, which was characterized by the infiltration of heterophils, macrophages, and lymphocytes with heterophilic exocytosis. Similar histological findings have been reported in hamsters infected with the variants of concern Gamma, Delta, and Omicron and the strain 614G [53]. Histologically, only mild cell death was observed, and quantification of cCAS3^+^ cells showed minimal apoptosis at 3 dpi. However, severe ciliary loss peaking at 3 dpi was detected by SEM investigation. The authors cannot exclude that a higher rate of cell death could have occurred at 2 dpi. Nevertheless, it has been described in hamster trachea and human bronchial epithelium, that cell death is limited, despite massive ciliary loss at various time-points following SARS-CoV-2 infection [31,54,55]. These results are in agreement with recently published data obtained from primary human bronchial cells and the hamster model during the early phase of infection [31]. The mentioned study showed that, in human cell cultures, ciliary loss is not associated with virally induced cell death, but rather, with the consequence of dedifferentiation of ciliated cells, which is associated with the loss of the transcription factor FOXJ1 at the mRNA and protein levels [31].

Loss of cilia and FOXJ1 protein expression has also been shown in samples from COVID-19 patients [56]. FOXJ1 is considered to be one of the master regulators of ciliogenesis in mammals and is regulated by TP73 in a complex transcriptional network [34,57,58]. Once basal cells exit the cell cycle and amplify the centrioles under the regulation of Cyclin Dependent Kinase Inhibitor 1A (Cdkn1a) and Myb, respectively, FOXJ1 is responsible for the apical docking of the centrioles to the apical membrane, which results in cilia production under the influence of TRAF3 Interacting Protein 1 (Traf3ip1) [58]. Loss of cilia during acute SARS-CoV-2 infection has been recently demonstrated in the hamster model as well, but no detailed investigation into the pathogenesis of the lesion has been performed [31]. Here, we show that the changes observed in the hamster trachea mirror alterations occurring in humans, including limited cell death and the loss of FOXJ1, confirming the value of the model to study the pathogenesis of COVID-19.

Our data further provide details on the ultrastructural features of ciliary loss and suggest a potential link between cilia retraction and virus entry. The main entry factors for SARS-CoV-2, ACE2, and TMPRSS2 are present on the surface of the respiratory cilia [32,38]. Accordingly, TEM investigations by others as well as the current authors have demonstrated that SARS-CoV-2 virions attach to the ciliary axonemes, as has been reported for other respiratory viruses [31,59]. However, there is no evidence that SARS-CoV-2 virions fuse with the ciliary plasma membrane to gain entry into the cytoplasm, as has been described for other viruses, e.g., the Sendai virus [60]. The original TEM description of respiratory coronavirus entry, provided by Afzelius, suggested that virions enter the cells through apical membrane invaginations between the cilia, from which they are taken up in small vesicles and transported to the Golgi apparatus [59]. He also observed that this process is accompanied by cilia internalization and suggested that the invaginations and vesicles mechanically displace basal bodies, which leads to the retraction of axonemes [59]. The results of our study and other recent publications [30,31] show similar morphologic features of virus entry and cilia retraction, including the presence of apical vesicles containing cilia and virions. However, the observation of a concurrent FOXJ1 loss argues against a purely mechanical dislocation of cilia by virions and suggests that the cilia retraction could be the consequence of FOXJ1 loss, which is triggered by infection. We suggest that it is indeed the loss of FOXJ1 that induces the dislocation of basal bodies [47] and that the associated membrane invaginations with virions are a secondary process. Similar vesicles around internalized cilia can also be observed in patients with a FOXJ1 mutation without association to viral particles [61]. However, it remains unclear whether SARS-CoV-2 directly induces a loss of FOXJ1 to increase the internalization of the cilia and promote virus uptake through membrane invagination or if the downregulation of FOXJ1 is an inflammation-induced protective host mechanism to prevent viral particles from interacting with receptors located on motile cilia. Previous results from in vitro studies using air liquid interface (ALI) cultures of fully differentiated human nasal epithelial cells (hNECs) confirmed that the origin of downregulation of ciliogenesis markers, including FOXJ1, could be antiviral state activation [20]. It was demonstrated that, in differentiating hNECs treated with synthetic imitators of viral components to simulate an antiviral state, the reduction of ciliated cells arises due to the downregulation of FOXJ1 and Tap73 [20]. Furthermore, interleukin-13 (IL-13) is implicated as playing a key role in the downregulation of FOXJ1 and the consecutive retraction of motile cilia infection [37,62,63]. IL-13 levels were proven to be upregulated in the upper respiratory tract in SARS-CoV-2-infected hamsters [64]. Hence, we assume that downregulation of FOXJ1 with retraction of motile cilia in the trachea of the golden Syrian hamster is more likely to be the result of a host-mediated inflammatory reaction induced by SARS-CoV-2 than a direct viral effect. Similar responses by ciliated epithelium have been described for other respiratory viruses, like paramyxoviruses and Influenza B, with some differences in the kinetics and onset of resolution [65,66]. In the initial stage, the retraction possibly contributes to the uptake of virions attached to cilia, but this seems to be an overall acceptable side effect, since virus replication and the resulting cell death are generally rapidly contained in the respiratory epithelium. Further investigations are needed to evaluate the causal relationship between virus entry and cilia retraction and to determine the cause of FOXJ1 loss.

Another interesting finding of the current work is the detection of virus particles in the intercellular space of the tracheal epithelium of hamsters infected with SARS-CoV-2. This finding suggests that SARS-CoV-2 also spreads via intercellular spaces. This interpretation of our results is reinforced by findings of a transiently impaired epithelial barrier function triggered by SARS-CoV-2 infection in an ALI model of primary human bronchial cells that would allow easier intercellular spread of the virus [31].

In addition to the detailed description of changes observed during acute infection with SARS-CoV-2, the results of the presented longitudinal study provide additional information on cilia regeneration following virus clearance and thereby the results of complement previous studies that focused on early changes. One of the most important findings is that the loss of FOXJ1 was not only observed at the time of active virus replication, but that the number of immunolabeled cells continuously decreased beyond viral clearance, indicative of an incomplete return to homeostasis. Another important result of the presented study is the demonstration of SARS-CoV-2-induced ultrastructural abnormalities in ciliary axonemes, which are indicative of secondary ciliary dyskinesia [37,47,61]. Ciliary dyskinesia is a possible sequel to various respiratory viral and bacterial infections and is associated with uncoordinated ciliary movement, resulting in impaired mucociliary clearance, which facilitates the development of secondary infections [22,23,24,25]. Interestingly, the alterations were not only observed during acute viral infection but also in regrown cilia at 14 dpi. Considering the importance of FOXJ1 for correct apical docking and the formation of cilia, the increased frequency of abnormal cilia is most likely related to the prolonged dysregulation of the transcription factor at the late time-point. The finding further underlines the incomplete and dysregulated regeneration of the respiratory epithelium following SARS-CoV-2 infection and suggests that secondary ciliary dyskinesia and a resulting increased susceptibility to respiratory infections could represent a potential long-term effect of COVID-19 infection [67,68,69]. Clinical evidence suggests that, in critically ill COVID-19 patients, ventilator-associated pneumonia and invasive fungal infections are commonly acquired coinfections, despite the use of broad-spectrum antibiotic treatment [70,71]. However, it remains debatable whether coinfections in COVID-19 patients are directly attributable to SARS-CoV-2 or whether they are a coincidental finding [72]. Additional studies are needed to determine the functional impact and clinical relevance of the structural changes observed in the presented study.

Finally, our work addressed the responses of basal cells to SARS-CoV-2 infection. Tracheal basal cell populations can rapidly respond to epithelial cell injury through proliferation and differentiation into ciliated cells [73,74,75]. It was previously shown that ciliary loss in SARS-CoV-2-infected hamsters is accompanied by the mobilization of CK5^+^ basal cells at 4 dpi [31]. Similarly, our data show an increased number of basal cells expressing CK14 peaking at 6 dpi. Subsequently, the number of CK14^+^ cells decreased but was still elevated at 14 dpi compared with in control animals. This finding indicates that the regeneration process could still ongoing at 14 dpi or that signaling pathways stimulating proliferation of CK14^+^ basal cells are still active. Interestingly, the number of p73^+^ cells showed slightly different kinetics over time. An increased number of p73^+^ cells was also detected during SARS-CoV-2-induced ciliary loss at 1 and 3 dpi. However, the number of p73^+^ cells was significantly decreased at 6 and 14 dpi compared with in the controls, in parallel to cilia regeneration. P73 has been reported to be expressed in basal cells to regulate their differentiation into different cell types [58]. Moreover, p73 also regulates FOXJ1 expression [58]. The cessation of p73^+^ cell proliferation at late time-points could be caused by the restoration of cilia, inducing termination of the stimulus for p73 upregulation in the basal cell pool.

The findings of the current study prompt an intriguing question regarding the origin of the cells regenerating cilia. Restoration of the intact ciliated respiratory epithelium can be theoretically achieved by the regeneration of sublethally injured cells with reactivation of the ciliogenesis machinery or by replacement from the basal cell pool. Although a mobilization of the basal cell pool was detected, the lack of FOXJ1^+^ cells suggests that at least some of the ciliated cells at 14 dpi could be redifferentiating cells that overcame viral infection. These cells appear to have a lasting perturbation of FOXJ1 but are still able to regenerate cilia, probably through the actions of alternative signaling pathways. Similar dysregulated surviving cells have been described and characterized in a mouse model of Influenza B infection [65]. Interestingly, these cells also showed the lasting downregulation of ciliogenesis genes including *Foxj1*, although the ultrastructural features of the cilia have not been investigated, unfortunately. It would be highly interesting to determine whether the cells with dysmorphic cilia are similar survivors of SARS-CoV-2 infection. An alternative explanation for the prolonged perturbation of ciliated cells is that reciliation is completely achieved by differentiating basal cells but that this differentiation is incomplete due to potentially persistent adverse external stimuli, e.g., inflammatory mediators. Additional studies involving the fate mapping of infected cells are needed to elucidate this question.

Overall, the dynamics of SARS-CoV-2-induced ciliary loss, FOXJ1 loss, and basal cell mobilization can be summarized by the hypothetical model depicted in the graphical abstract. Acute infection causes a progressive decline in FOXJ1 and retraction of the cilia into the cytoplasm, which co-occurs with membrane invaginations and the formation of vesicles that facilitate virus entry into the cell. Cell death and sloughing are minimal, but ciliary loss triggers the mobilization of basal cells, which ceases when cilia are restored after two weeks. Cilia are completely regrown at 14 dpi, but the process does not appear to be fully functional, since prolonged loss of FOXJ1 and ultrastructural features of secondary ciliary dyskinesia were observed.

In conclusion, our work complements previous findings regarding the effects of SARS-CoV-2 infection on the integrity of the ciliated epithelium by providing a longitudinal study design and a thorough investigation of the cilia regeneration process following virus clearance, an area that was only partially addressed in previous publications [29,31,76]. Importantly, this investigation revealed that the cilia regeneration process appears to be dysregulated and that full restoration of the normal ultrastructure is not achieved after 2 weeks. Particularly the long-lasting loss of FOXJ1 and the morphologic indicators of secondary ciliary dyskinesia represent novel observations that add value to the research field and require future detailed investigations.

## 4. Materials and Methods

### 4.1. Animal Experiments

All animal experiments were performed according to directive 2010/63/EU of the EU legislation and were approved by the local authorities Behörde für Justiz und Verbraucherschutz der Freien und Hansestadt Hamburg, Department for Lebensmittelsicherheit und Veterinärwesen (protocol code N032/2020 22 April 2020). Eight to ten week old female and male golden Syrian hamsters (Janvier Labs, Le Genest-Saint-Isle, France) were housed in groups of 2 to 4 individuals in isolated ventilated cages under standardized conditions (21 ± 2 °C, 40–50% relative humidity, 12 h light/dark cycle, food, and water ad libitum) at the Leibniz Institute for Experimental Virology in Hamburg, Germany, as previously published [42]. Intranasal inoculation with 10^5^ plaque-forming units (p.f.u.) of SARS-CoV-2 (SARS-CoV-2/Germany/Hamburg/01/2020; ENA study PRJEB41216 and sample ERS5312751) [77] or phosphate-buffered saline (PBS, control), respectively, was performed under general anesthesia, as described (Supplementary Figure S2) [43]. At 1, 3, 6, and 14 days post-infection (dpi), ten animals per group were euthanized, and tracheal tissue was collected and fixed in 10% neutral-buffered formalin (Chemie Vertrieb GmbH & Co Hannover KG, Hannover, Germany) or 5% glutaraldehyde (Merck KGaA, Darmstadt, Germany) [43].

### 4.2. Histology

For histological examination, tracheal samples were formalin-fixed and paraffin embedded (FFPE), processed in 2-μm-thick serial sections, and stained with hematoxylin and eosin (H&E). Slides were scanned using a Hamamatsu NanoZoomer S210 Digital slide scanner (Hamamatsu), and respiratory epithelium in the trachea was evaluated in a bright field at a magnification of up to 400×. Tracheal inflammation was quantified using a semiquantitative scoring system. Tracheal sections were assessed by separate scoring of the degree and distribution of inflammatory cell infiltration. The degree of inflammatory cell infiltration was graded with as (1) minimal (single cells), (2) mild (1–2 layers), (3) moderate (3–4 layers), (4) severe (5–6 layers), and (5) massive (>7 layers). The distribution of inflammation was assessed as follows: (0) no lesion (0% of tissue affected), (1) minimal (<1% of tissue affected), (2) mild (2–25% of tissue affected), (3) moderate (26–50% of tissue affected), (4) marked (51–75% of tissue affected), and (5) subtotal (>75% of tissue affected).

The overall inflammation score (0–25) was calculated for each animal by multiplying the score of degree of inflammation (0–5) with the score of the distribution of the inflammatory cell population (0–5).

Separate overall scores were assigned for each tracheal section. If multiple sections of tracheal epithelium were available for one animal, the means of the individual overall scores were calculated per animal. Histopathological semiquantitative evaluations were performed in a blinded manner by veterinary pathologists (TS, LA) and subsequently confirmed by a European-board-certified veterinary pathologist (WB, MC).

### 4.3. Immunohistochemistry

Immunohistochemistry was performed using the avidin-biotin-peroxidase complex (ABC) method (Vector Laboratories, Burlingame, CA, USA) and 3,3′-Diaminobenzidine tetrahydrochloride (Sigma-Aldrich, St. Louis, MO, USA) or the Dako EnVision+ polymer system (Dako Agilent Pathology Solutions, CA, USA), as described previously [78,79,80]. Details about the applied antibodies can be found in Table 1. For negative controls, specific primary antibodies were replaced by ascitic fluid from nonimmunized BALB/cJ mice (for p73 and SARS-CoV-2 NP) and serum from nonimmunized rabbits (cCaspase-3, CK14, and FOXJ1).

### 4.4. Scanning Electron Microscopy

For scanning electron microscopy (SEM), tracheal samples were fixed in 5% glutaraldehyde (Merck KGaA, Darmstadt, Germany) buffered with 0.1 M cacodylate buffer (Serva Electrophoresis GmbH, Heidelberg, Germany) and subsequently embedded by a modified osmium (O)-thiocarbohydrazide (T)-embedding (OTOTO) (Roth C. GmbH & Co. KG, Karlsruhe, Germany) protocol. After critical-point-drying, the ventral part of the trachea was mounted on 0.5′’ Aluminium Specimen Stubs (Agar Scientific, Stansted, United Kingdom) using 12 mm Leit-Tabs (Plano GmbH, Wetzlar, Germany) and Conductive Carbon Cement after Göcke (Plano GmbH, Wetzlar, Germany) sputter-coated with gold (SCD40, Oerlikon Balzers, Balzers, Liechtenstein), as previously described. [81] Samples were examined using a Zeiss EVO 15 scanning electron microscope (Carl Zeiss Microscopy GmbH, Jena, Germany) operating at 10 kV.

Examination of the tracheal respiratory epithelium was performed in a blinded manner by assessing the available epithelium and selecting 4 representative localizations per sample. For each of the 4 localizations, one picture was generated at 4000× magnification. Numbers of multiciliated cells and nonciliated cells were counted manually with ImageJ, and the median percentage of ciliated cells was calculated for each animal. For the illustration, pictures were manually colorized with Adobe Photoshop^®^ (Adobe Inc., San José, CA, USA), depicting motile cilia in red, nonciliated areas in yellow, and unidentifiable materials, e.g., mucus, erythrocytes, and debris, in blue.

### 4.5. Transmission Electron Microscopy

Glutaraldehyde-fixed tracheal tissue was rinsed for one night in cacodylate buffer (Serva Electrophoresis GmbH, Heidelberg, Germany). Post-fixation was conducted using 1% osmium tetroxide (Roth C. GmbH & Co. KG, Karlsruhe, Germany), followed by dehydration in graded series of alcohol. Thereafter, samples were embedded in epoxy resin, as previously described [82]. Ultrathin sections were cut from representative localizations of the ciliated epithelium, contrasted with uranyl acetate and lead citrate, and evaluated on a transmission electron microscope (EM 10C, Carl Zeiss Microscopy GmbH, Jena, Germany). To quantify ultrastructural alterations indicative of secondary ciliary dyskinesia, pictures of transversal sections of ciliary axonemes were taken. A total of 100 axonemes were evaluated for each animal. Ciliary alterations including compound cilia of the bulging and adhesive types as well as ciliary blebs were quantified and percentages were calculated. Schematic illustrations of ciliary alterations were generated using Apple Keynote 6.6.2 for MacOS.

### 4.6. Digital Image Analyses

For the quantification of immunolabeled cells in tracheal tissue, slides were digitized using the Hamamatsu NanoZoomer S210 (Hamamatsu Phototonics. Hersching am Ammersee, Germany) slide scanner. Image analysis was performed using the open-source software package QuPath for the digital pathology image analysis [83]. The tracheal epithelium was indicated as a region of interest (ROI) by veterinary pathologists (GB, FA) and the total numbers of immunolabeled and nonlabeled cells were determined by the automated analysis of ROIs, based on marker specific thresholding.

### 4.7. Statistical Analyses

Statistical analyses were performed using SPSS for WindowsTM v. 27 (IBM^®^ SPSS^®^ Statistics, SPSS Inc., Chicago, IL, USA). Data were analyzed for normality using the Shapiro–Wilk test. Significant differences within groups (SARS-CoV-2; control) were investigated using one-tailed Mann–Whitney U tests. Statistical significance was accepted at *p*-values of ≤0.05 (*), ≤0.01 (**), and ≤0.001 (***). Graphs were designed using GraphPad Prism (GraphPad Software, San Diego, CA, USA) for Windows™.

## Figures and Tables

**Table 1:** Primary antibodies, dilutions, detection systems, and pretreatments for immunohistochemistry. cCaspase-3: cleaved caspase 3; CK: cytokeratin; MW: microwave; SARS-CoV-2 NP: severe acute respiratory syndrome coronavirus-2 nucleoprotein.

Antigen	Pretreatment	Dilution	Clonality	Supplier	Catalog Number	Positive Control
cCaspase-3	MW 800 W—Citrate Buffer pH 6.0	1:500	Monoclonal rabbit, clone 5A1E	Cell signaling	9664	Lymphoid tissue
CK14	MW 800 W—Citrate Buffer pH 6.0	1:800	Polyclonal rabbit	Invitrogen	PA5-16722	Liver and esophagus
FOXJ1	MW 800 W—Citrate Buffer pH 6.0	1:300	Polyclonal rabbit	LSBio	LS-B951	Trachea
p73	MW 800 W—Citrate Buffer pH 6.0	1:1000	Monoclonal mouse, clone 5B1288	Novusbio	NB 100-56674SS	Trachea
SARS-CoV-2 NP	MW 800 W—Citrate Buffer pH 6.0	1:16000	Monoclonal mouse, clone 5	SinoBiological	40143-MM05	SARS-CoV-2 infected lung

## Data Availability

The data that support the findings of this study are available from the corresponding author upon reasonable request.

## Supplementary Materials

The following supporting information can be downloaded at: https://www.mdpi.com/article/10.3390/ijms23095124/s1.
