# Supplementary material for: SARS-CoV-2 Infection Dysregulates Cilia and Basal Cell Homeostasis in the Respiratory Epithelium of Hamsters
Source: Int J Mol Sci. 2022 May 4;23(9):5124. doi: 10.3390/ijms23095124 (PMC9102945; doi:10.3390/ijms23095124)
Supplement: Supplementary file 1 [file ijms-23-05124-s001.zip › ijms-1691482-supplementary 2.pdf]

International Journal of *Molecular Sciences*

MDPI

Article

SARS-CoV-2 Infection Dysregulates Cilia and Basal Cell Homeostasis in the Respiratory Epithelium of Hamsters

Tom Schreiner[1,2,†], Lisa Allnoch[1,2,†], Georg Beythien[1], Katarzyna Marek[1,2], Kathrin Becker[1], Dirk Schaudien[3], Stephanie Stanelle-Bertram[4], Berfin Schaumburg[4], Nancy Mounogou Kouassi[4], Sebastian Beck[4], Martin Zickler[4], Gülsah Gabriel[4], Wolfgang Baumgärtner[1,2,‡,*], Federico Armando[1,‡], Malgorzata Ciurkiewicz[1,‡]

1 Department of Pathology, University of Veterinary Medicine Hanover Foundation, 30559 Hanover, Germany
2 Center for Systems Neuroscience (ZSN), University of Veterinary Medicine Hanover Foundation, 30559 Hanover, Germany
3 Fraunhofer Institute for Toxicology and Experimental Medicine, 30625 Hanover, Germany
4 Department for Viral Zoonoses-One Health, Leibniz Institute for Experimental Virology (HPI), 20251 Hamburg, Germany
* Correspondence: Wolfgang.Baumgaertner@tiho-hannover.de
† These authors contributed equally to this work as first authors
‡ These authors contributed equally to this work as last authors

Supplementary material

Citation: Schreiner, T.; Allnoch, L.; Beythien, G.; Marek, K.; Becker, K.; Schaudien, D.; Stanelle-Bertram, S.; Schaumburg, B.; Kouassi, N.M.; Beck, S.; et al. SARS-CoV-2 Infection Dysregulates Cilia and Basal Cell Homeostasis in the Respiratory Epithelium of Hamsters. *Int. J. Mol. Sci.* **2022**, *23*, 5124. https://doi.org/10.3390/ijms23095124

Academic Editors: Malgorzata Kloc and Jacek Z. Kubiak

Received: 5 April 2022
Accepted: 3 May 2022
Published: 4 May 2022

Publisher's Note: MDPI stays neutral with regard to jurisdictional claims in published maps and institutional affiliations.

Copyright: © 2022 by the authors. Licensee MDPI, Basel, Switzerland. This article is an open access article distributed under the terms and conditions of the Creative Commons Attribution (CC BY) license (https://creativecommons.org/licenses/by/4.0/).

This file includes:

Fig. S1.

Fig. S2

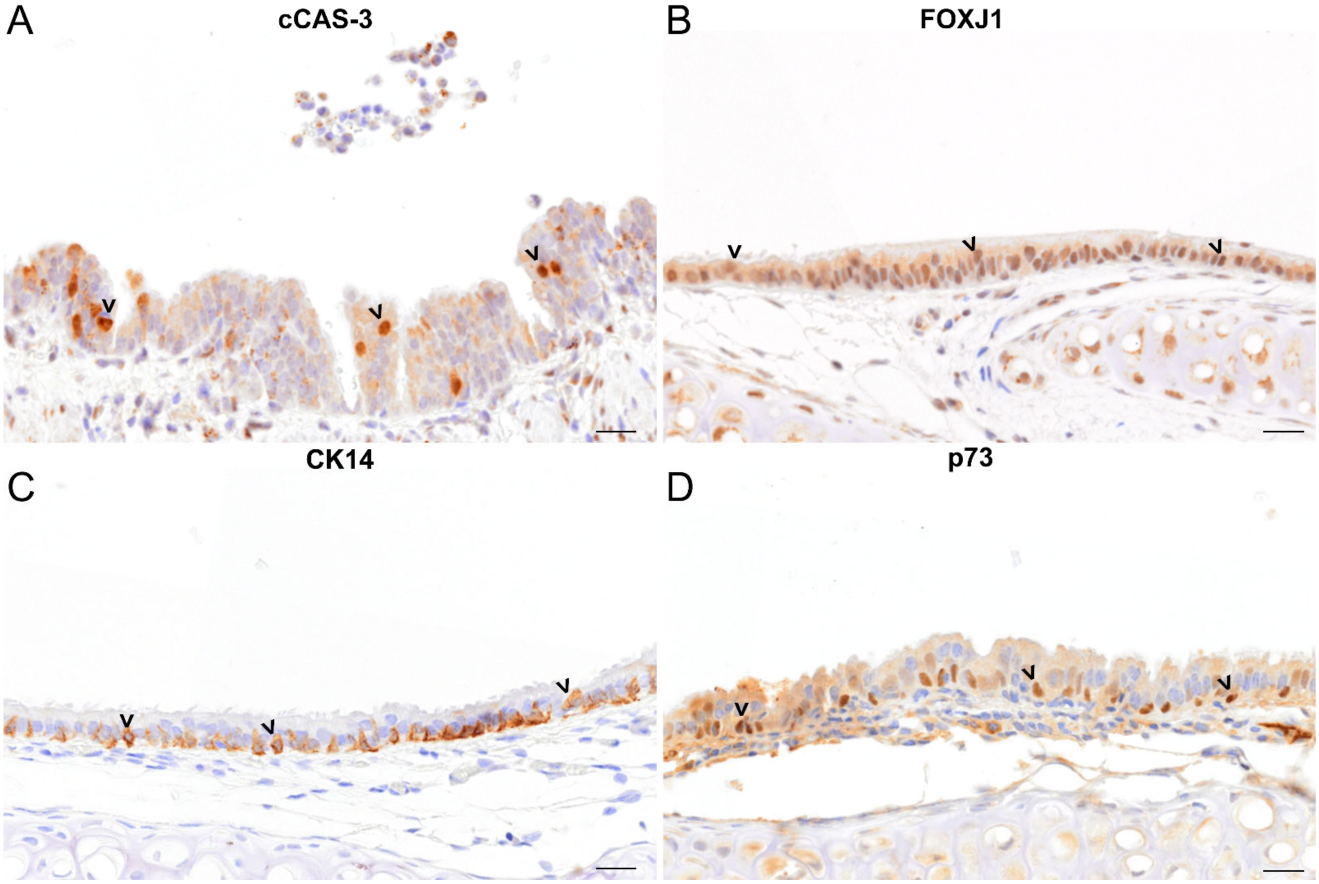

Supplementary Figure S1. Immunohistochemistry for the markers of apoptosis, differentiated ciliated cells and basal cells. Representative images of cleaved caspase-3 (**A**, cCAS-3, marker for apoptosis, cytoplasmic signal), Forkhead box protein J1 (**B**, FOXJ1, transcription factor and marker for differentiated ciliated cells, nuclear signal), cytokeratin 14 (**C**, CK14, marker for basal cells, cytoplasmic signal), and p73 (**D**, marker for basal cells, nuclear signal) in respiratory epithelium of hamsters. Examples of immunolabelled cells are indicated by arrowheads. Scale bars: 50 µm.

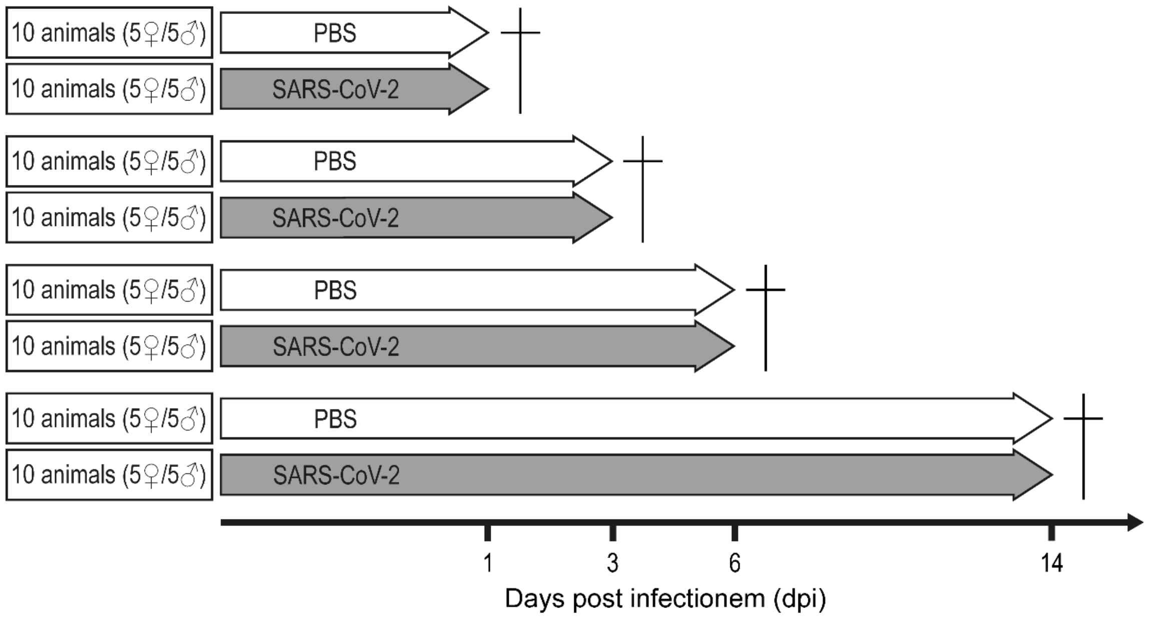

Supplementary Figure S2. Animal experiment. 10 golden Syrian hamsters per group were intranasally inoculated with severe acute respiratory syndrome coronavirus (SARS-CoV-2) (SARS-CoV-2/Germany/Hamburg/01/2020; ENA study PRJEB41216 and sample ERS5312751) or phosphate-buffered saline (PBS), respectively. SARS-CoV-2 and PBS injected/inoculated animals were euthanized at 1, 3, 6 or 14 dpi.
